# Healthy Gut Microbiome Composition Enhances Disease Resistance and Fat Deposition in Tibetan Pigs

**DOI:** 10.3389/fmicb.2022.965292

**Published:** 2022-07-19

**Authors:** Peng Shang, Mingbang Wei, Mengqi Duan, Feifei Yan, Yangzom Chamba

**Affiliations:** ^1^College of Animal Science, Tibet Agriculture and Animal Husbandry University, Linzhi, China; ^2^The Provincial and Ministerial Co-founded Collaborative Innovation Center for R&D in Tibet Characteristic Agricultural and Animal Husbandry Resources, Linzhi, China

**Keywords:** Tibetan pig, Yorkshire pig, gut microbiota, 16SrRNA gene, disease resistance, fat deposition, microbiome composition

## Abstract

The gut microbiota is involved in a range of physiological processes in animals, and modulating the microbiome composition is considered a novel target for identifying animal traits. Tibetan pigs show better fat deposition and disease resistance compared to Yorkshire pigs. However, studies investigating the correlation between favorable characteristics in Tibetan pigs and the gut microbial community remain scarce. In the current study, 1,249,822 high-quality sequences were obtained by amplicon sequencing of the colon contents of Tibetan and Yorkshire pigs. We found that at the boundary level, the abundance and relative abundance of colon bacterial community in Tibetan pigs were higher than that in Yorkshire pigs (*P* > 0.05). Phylum level, Firmicutes were the dominant colonic microflora of Tibetan and Yorkshire pigs, and the ratio of Firmicutes to Bacteroides in Tibetan pigs was slightly higher than in Yorkshire pigs. Actinobacteria and Spirobacteria were significantly higher in Tibetan pigs than in Yorkshire pigs (*P* < 0.05). At the genus level, the relative abundance of Bifidobacterium, *Lactobacillus*, and Bacteriologist, which are related to disease resistance, was significantly higher than that in Yorkshire pigs in Yorkshire pigs. In conclusion, the composition and abundance of colonic intestinal microflora in Tibetan pigs were closely related to their superior traits. Bifidobacteria, Ruminococcaceae, and Family-XIII-AD3011-Group are conducive to improving disease resistance in Tibetan pigs. *Lactobacillus* and *Solobacterium* were observed to be the main bacterial communities involved in fat deposition in Tibetan pigs. This study will provide a new reference for the development and utilization of Tibetan pigs in future.

## Introduction

The Tibetan pig is an indigenous fatty pig breed in China, mainly found in Tibet and the Sichuan, Gansu, and Yunnan provinces, where the altitude is approximately 3,000 m above sea level or higher (Ma et al., 2019). Tibetan pigs are the only high-altitude pasture pig breed in China, and live in high-altitude and cold areas; these pigs are characterized by strong fat deposition ability, disease and stress resistance, resistance to low oxygen conditions, and tolerance to rough feeding (Ai et al., 2014; Zhang et al., 2017; Shang et al., 2019). The Yorkshire pig is a typical lean pig breed that originated in the United Kingdom. It is widely distributed and is currently one of the most commonly raised pig breeds worldwide. Yorkshire pigs have excellent characteristics such as fast weight gain, high feed conversion rate, and high lean meat rate of carcasses (Gong et al., 2022). At present, Tibetan pigs on the Tibetan plateau are raised mainly through stabling and half-stabling feeding, often grazing in the sports arena, and their feed, comprising grass, leaves, fruits, roots, and insects, is rich in fiber. Therefore, the special living environment and half-barn feeding method make the Tibetan pig disease-resistant and they show excellent characteristics of fatty deposits.

The intestinal tract is the main site of nutrient digestion and absorption. Intestinal microbes are dense bioactive communities that serve as the junction between animals and their nutritional environment (Anand and Mande, 2018). Thus, their activity profoundly affects many aspects of host animal physiology and metabolism (Judkins et al., 2020). Intestinal microbiota is essential for nutrient digestion and absorption, and plays an important role in the physiological, nutritional, and immune functions of the host (Park et al., 2014; Chen et al., 2017). The intestinal mucosa and microbial community together promote the development of the host immune system. Symbiotic microorganisms affect disease resistance in animals by competing for receptors and intestinal nutrients, producing antibacterial compounds, creating a disease-resistant microenvironment, and stimulating the innate immune system (Fernandez et al., 2003; Liang et al., 2014).

The gastrointestinal tract of pigs contains numerous species of bacteria, the composition and relative proportions of which vary with animal species, age, nutrition, and environmental factors (Lu et al., 2014; Yang et al., 2014). To date, a series of intestinal microbial structural components and metabolites have been found to interact directly with host intestinal cells and tissues, often by consuming, storing, and redistributing energy to maintain the dynamic balance of the body (Hillman et al., 2017). It affects nutrient absorption and host health (Ghosh et al., 2021; Gill et al., 2021). The mechanisms of microbial influence are mainly derived from microbial activity in the gut, and then projected into the body through a variety of integrated pathways. The complexity of these interactions means that different microbial community compositions can lead to different results, which may be related to the host diet or a specific system. It has also been shown to be closely related to the host species, genetic background, and intestinal microbial taxa and characteristics of the host (Kim and Isaacson, 2015).

The colon is the main site of microbial fermentation and the core flora in the gut directly affects intestinal function (Luo et al., 2021). Recently, the intestinal microbiota of Tibetan pigs has been extensively studied; however, the relationship between the composition of colon microbiota and lipid deposition and the host resistance to disease requires worth further exploration. In this study, 16SrRNA high-throughput sequencing technology was used to compare the specificity of the colon microbial structure and composition of Tibetan pigs and Yorkshire pigs and to explore the effects of the colon microbial community on disease resistance and fat deposition traits of Tibetan pigs. This will be conducive to further development and utilization of Tibetan pig germplasm resources.

## Materials and Methods

### Sample Collection

The samples in this study were randomly collected from the practice pasture of the Tibet Agriculture and Animal Husbandry University, Linzhi, Tibet (average altitude 2,980 m above sea level, longitude 94.34°E, latitude 29.67°N). Six adult Tibetan pigs (T1, T2, T3, T4, T5, and T6) and six Yorkshire pigs (Y1, Y2, Y3, Y4, Y5, and Y6) were used, both male and female. Yorkshire and Tibetan pigs were fed using the traditional and half-house feeding methods, respectively. In addition to the feed, which was the same as that provided to the Yorkshire pigs, Tibetan pigs also ate fruit, grass, leaves, roots, and other food. The pigs were sacrificed by bloodletting the anterior vena cava. The abdominal cavity was cut open, the intestine was removed, and the 20 cm intestine was ligated in the middle part of the colon. Under aseptic conditions, a small opening was made in the middle of the ligated intestine with ophthalmic scissors, squeezed into an aseptic frozen tube, placed into liquid nitrogen for quick freezing, and the sample was stored at −80°C until subsequent use and further 16SrRNA analysis. Colon samples were collected and immediately placed in formalin for histopathological analyses.

### Histological Analysis

The collected colon samples were placed at room temperature and fixed for 24 h. After the fixed colon was dehydrated in increasing ethanol concentration and cleared in xylene, paraffin was embedded to prepare histological sections of 5 mm thickness. Sections of 5 mm were stained with hematoxylin for 3 min, and then stained with eosin at room temperature for 20 s. Sections were examined by inverted microscope (OlympusBX51, Japan), the morphology of colon was observed.

### DNA Extraction and 16SrDNA Amplicon Sequencing

A Hi Pure Stool DNA Kit (model D3141, Guangzhou Meiji Biotechnology Co., Ltd., Guangzhou, China) was used to extract microbial DNA. The purity and concentration of DNA were determined using Namedrop 2000 (Mother). The integrity of the DNA was detected using 1.0% agarose gel electrophoresis. To investigate the gut microbial composition, the V3–V4 region of 16SrDNA was amplified by PCR with primers 341F (CCTACGGGNGGCWGCAG) and 806R (GGACTACHVGGGTATCTAAT). As mentioned earlier, triple polymerase chain reaction was carried out (procedure: 95°C, 2 min; 98°C, 10 s; 62°C, 30 s; 68°C, 30 s; 27 cycles, 68°C, 10 min; system: 5 μL 10 × KOD buffer, 5 μL 2.5 mM dNTPs, upstream and downstream primers 1.5 μL, 1 μL KOD polymerase, and 100 ng template DNA). According to the manufacturer’s instructions, the amplification products were extracted on a 2% agarose gel, and amplification products obtained on the second round were purified using AMPure XP Beads. All amplification products were quantified using an ABI Step One Plus Real-Time PCR System (Life Technologies, CA, United States), and the pooling was sequenced according to the PE250 mode of Novaseq 6000.

### Bioinformatics and Statistical Analysis

Adapters and low-quality raw data may influence the assembly and analysis of data. To obtain high-quality clean readings, the original readings were further filtered according to the guidelines of FASTP^1^ to remove reads containing 10% unknown nucleotides and to remove less than 80% of the bases with mass (Q) > 20. Subsequently, FLASH (version 1.2.11) (Magoč and Salzberg, 2011) was used to merge the paired-end clean readout into the original label, with a minimum overlap of 10 bp and a mismatch error rate of 2%. The interference sequences of the original tags were filtered through the QIIME (version 1.9.1) (Caporaso et al., 2010) pipeline under specific filtering conditions (Bokulich et al., 2013) to obtain high-quality, clean tags. The cleaning tags were searched against the reference database^2^. Reference-based chimera examination was performed using the UCHIME algorithm^3^. Following this, all chimeric tags were removed, and valid tags were obtained and employed for further analysis. UPARSE (Edgar, 2013) pipes were used to aggregate valid labels into ≥97% operational taxa (OTU). The tag sequence with the highest abundance was selected as the representative sequence in each cluster. Based on the SILA database^4^ (Pruesse et al., 2007), the RDP classifier (version 2.2) (Wang et al., 2007) was used to classify the representative sequences using a naive Bayesian model. The confidence threshold was 0.8–1.0.

The abundance statistics for each category were visualized using Krona (version 2.6) (Ondov et al., 2011). The stacked bar chart of community composition was visualized using the R Project ggplot2 package (version 2.2.1). The diversity indexes of Chao1, Simpson, and Alpha were calculated using QIIME. The ecological function spectrum of the bacteria was generated using the Functional Annotation of Prokaryotic Taxa (FAPROTAX) and related software (version 1.0) (Louca et al., 2016). Tax4 Fun (version 1.0) or PICRUSt (version 2.1.4) were used to analyze the KEGG path of OTUs (version 1.0) (Aßhauer et al., 2015).

### Statistical Analysis

The abundance statistics for each classification were visualized using Krona. The diversity indices of Chao1, Simpson, and Alpha were calculated using QIIME. Welch’s *t*-test and Wilcoxon rank test were used for alpha diversity analysis. The ecological function map of the bacteria was generated using the FAPROTAX database and related software (version 1.0). The functional differences between groups were tested using Welch’s *t*-test, Wilcoxon rank test, Kruskal-Wallis *h* test, and Tukey’s honest significant difference (HSD) test.

## Results

### Differences in Colonic Morphology Between Tibetan and Yorkshire Pigs

**FIGURE 1:**
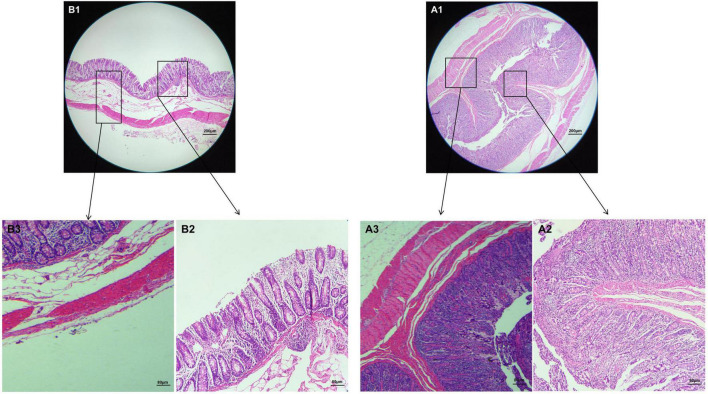
Morphological observation of colon in Tibetan pigs and Yorkshire pigs under different magnifications. **(A1–A3)** Colon and intestinal sections of Tibetan pigs; **(B1–B3)** Colon and intestinal sections of Yorkshire pigs.

The results of HE staining showed that the intestinal structure of Tibetan pig and Yorkshire pig was intact, the boundary was clear, and the goblet cells were evenly distributed in the intestinal mucosa. As can be seen from Figures 1A1,B1, the colonic intestines of Tibetan pigs and Yorkshire pigs of the same age are generally smaller than those of Yorkshire pigs, and the intestinal structure is more compact. Under the same magnification, the colon morphology of Tibetan pig and Yorkshire pig showed that compared with Yorkshire pig, the thickness of colon mucosal layer, intestinal villus density, intestinal villus length and muscle layer thickness of Tibetan pig were larger than those of Yorkshire pig (Figure 1).

### Sequence Analysis

The results of this study showed that a total of 1,249,822 high quality sequences were available from 12 fecal samples, and the average effective combination sequence of each sample was 104,151. The length distribution of each sample was 200∼474 bp (Table 1). All the optimized sequences were compared with the OTU representative sequences using the UPARSE software, and sequences with more than 97% similarity to the representative sequences were selected to generate OTUs. After classification and matching, a total of 16,070 OTUs were obtained.

**TABLE 1:** Quantitative statistics of Tags and OTUs.

Samples name	Raw reads	Clean reads	Raw tags	Clean tags	Chimera	Effective tags	Effective ratio (%)	OTUs
T1	114113	114020	112733	112027	14456	97571	85.5	642
T2	130597	130468	129126	128134	16688	111446	85.34	1075
T3	129466	129353	127884	126912	18900	108012	83.43	1182
T4	121107	120999	119507	118919	14279	104640	86.4	1142
T5	133281	133178	131648	130686	20262	110424	82.85	1694
T6	126212	126107	124344	123082	16141	106941	84.73	1532
Y1	130924	130834	129459	127823	19308	108515	82.88	1578
Y2	133586	133461	131642	129998	18054	111944	83.8	1451
Y3	120997	120894	119640	118413	17412	101001	83.47	1547
Y4	130559	130456	128942	127444	17529	109915	84.19	1197
Y5	121377	121280	119809	118613	15490	103123	84.96	1042
Y6	88623	88546	87511	87022	10732	76290	86.08	752

### Analysis of Microbial Composition and Structure

**FIGURE 2:**
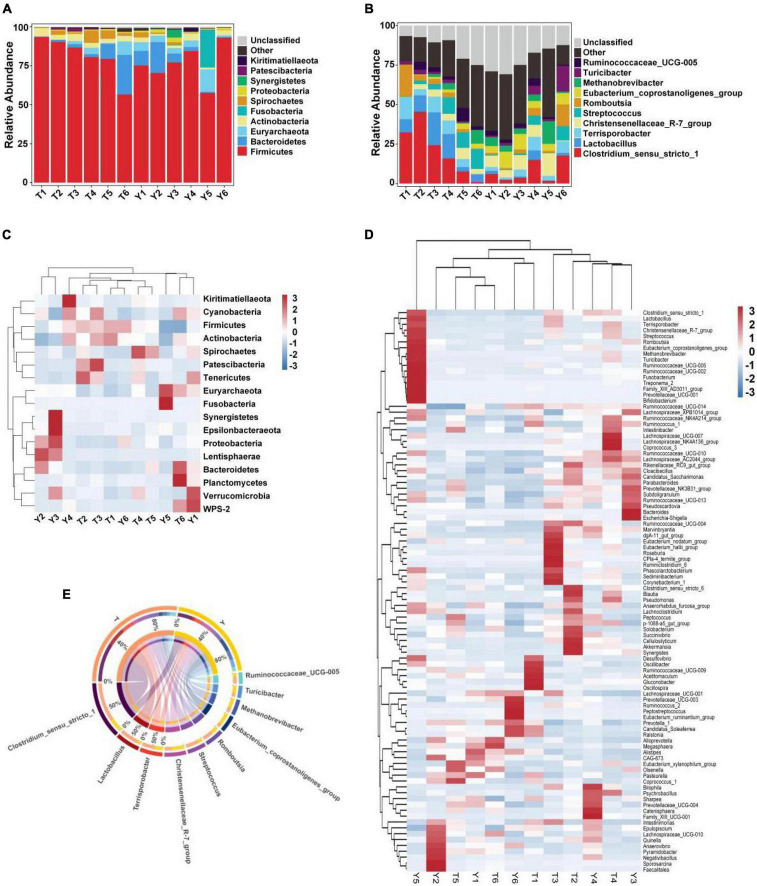
Heat map showing the relative abundance of intestinal microflora in Tibetan and Yorkshire pigs at phylum and genus level. **(A,B)** Represent the distribution at the phylum and genus level, respectively. **(C)** Heat maps of the 17 most common gates in different communities. **(D)** Heat maps of the 96 most common genera in different communities. Each color block in the heat map represents the relative abundance of a genus in the sample. Clustering can distinguish taxon with different abundance, and color gradient and similarity can reflect the similarities and differences of multiple samples at different classification levels. The blue-red gradient shows the change of abundance from low to high. **(E)** Composition of microorganisms among horizontal species.

The relative abundance of taxa at the phylum and genus levels was evaluated based on the distribution of microbial taxa in the two groups (Figure 2). The abundant microflora in the intestinal tract of Tibetan pigs and Yorkshire pigs show great diversity at both the gate and genus levels. At the gate level, Firmicutes, Bacteroidetes, Euryarchaeota, Actinobacteria, Fusobacteria, Spirochetes, Proteobacteria, Synergistetes, Patescibacteria, and Kiritimatiellaeota were the ten most abundant phyla (Figure 2A). At the genus level, *Clostridium sensu stricto 1*, *Lactobacillus*, *Terrisporobacter*, *Christensenellaceae* R-7 group, *Streptococcus*, *Romboutsia*, *Eubacterium coprostanoligenes_group*, *Methanobrevibacter*, *Turicibacter*, and *Ruminococcaceae* UCG-005 were the ten most abundant genera (Figure 2B). The horizontal cluster analysis of phyla and genera using a heat map showed that 17 phyla were co-clustered at the gate level, 97 different genera were co-clustered at the genus level, and the distribution of bacterial phyla and genera in different individuals was consistent with the relative abundance stack map. The similarity of intra-group samples was also shown to be higher than that of inter-group samples (Figures 2C–E).

**FIGURE 3:**
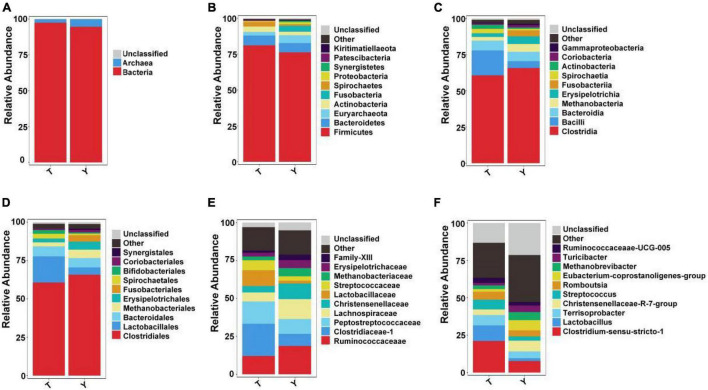
Ten most abundant taxa of intestinal microflora in Tibetan (T) and Yorkshire (Y) pigs at boundary. Relative abundance at the kingdom **(A)**, phylum **(B)**, class **(C)**, order **(D)**, family **(E)**, and genus **(F)** levels.

Upon studying the classification and distribution of the microbial communities in the two groups, the relative percentages of the dominant taxa at the boundaries, phyla, classes, orders, families, and genera were evaluated (Figure 3). More than 94.5% of the colonic microorganisms in Tibetan and Yorkshire pigs belong to the bacterial kingdom, and the proportion of colonic microorganisms in Tibetan pigs (97.44%) was larger than that in Yorkshire pigs (94.54%) (Figure 3A). At the gate level, the thick-walled bacteria in the colons of Tibetan pigs and Yorkshire pigs were the dominant communities, accounting for 81.15 and 76.26%, respectively (Figure 3B). The 10 most prevalent colonic microorganisms in Tibetan and Yorkshire pigs were *Clostridia* (60.70 and 65.74%), *Bacilli* (17.17 and 4.79%), *Bacteroidia* (6.80 and 6.44%), *Methanobacteria* (3.06 and 5.43%), *Erysipelotrichia* (2.78 and 5.27%), *Fusobacteria* (2.54 and 4.16%), *Spirochaetia* (2.54 and 1.03%), *Actinobacteria* (2.10 and 0.92%), *Coriobacteriia* (0.92 and 1.55%), and *Gammaproteobacteria* (0.91 and 0.98%) (Figure 3C). The relative abundance of colonic *Bacilli* and *Spirochaetia* in Tibetan pigs was significantly higher than that in Yorkshire pigs. *Clostridiales* were dominant in the colons of Tibetan and Yorkshire pigs, accounting for more than 60% of the total community composition (Figure 3D). The relative abundances of *Lactobacillus* (17.01%), *Spirulina* (3.06%), and *Bifidobacterium* (2.43%) in the colonic microbiota of Tibetan pigs were significantly higher than those of Yorkshire pigs (4.76%, 1.03%, and 0.79%) (*P* < 0.05). The composition of microflora at the family level is shown in Figure 3E. The relative abundances of *Clostridium*-1 (21.26%), *Enterostreptococcus* (14.67%), *Lactobacillus* (10.44%), and *Streptococcus* (6.50%) in the colonic microbiota of Tibetan pigs were higher than those of Yorkshire pigs (*Clostridium*-17.94%, digestive *Streptococcaceae* 9.75%, *Lactobacillaceae* 1.83%, and *Streptococcus* 2.90%). Figure 3F shows the composition of the microflora at the genus level. Predominantly, *Clostridium sensu stricto 1*, *Lactobacillus*, *Terrisporobacter*, *Christensenellaceae* R-7 group, *Streptococcus*, *Romboutsia*, *Eubacterium coprostanoligenes* group, *Methanobrevibacter*, *Turicibacter*, and *Ruminococcaceae*_UCG-005 were observed. The predominant groups in the Tibetan and Yorkshire pigs were substantially different.

### Analysis of Colonic Microbial Diversity in Tibetan and Yorkshire Pigs

**FIGURE 4:**
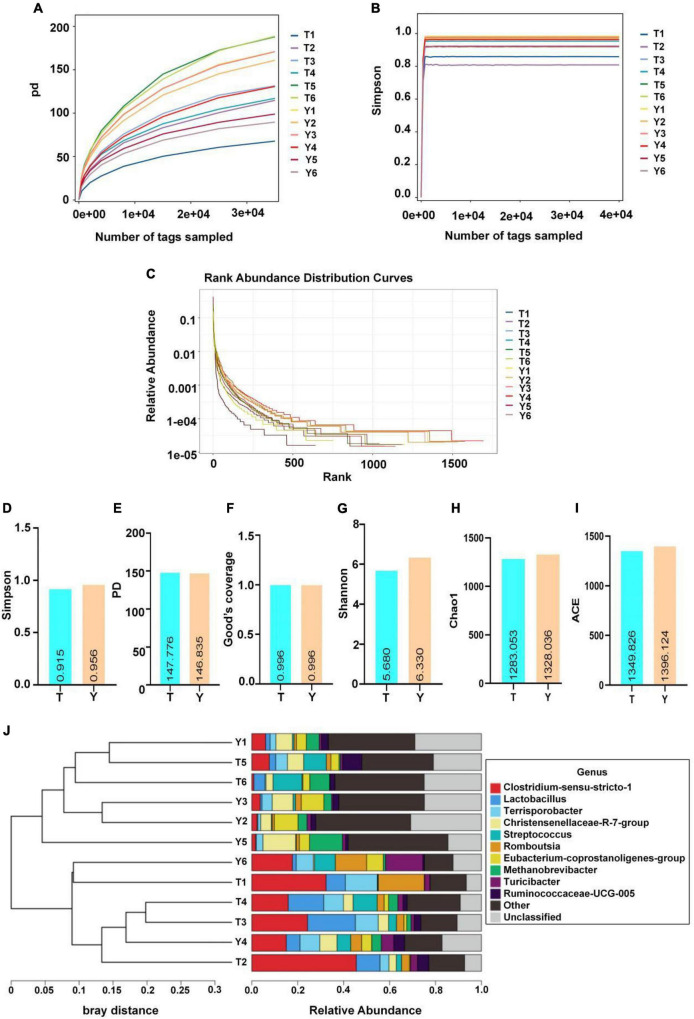
Microbial diversity in colon of Tibetan (T) and Yorkshire (Y) pigs. **(A)** PD diversity index curve. **(B)** Simpson diversity index curve. **(C)** Rank abundance curve. **(D–I)** Alpha diversity index (Simpson, PD, Good’s coverage, Shannon, Chao1, and ACE). **(J)** UPGMA cluster tree. Each curve represents a sample.

The sequence numbers were confirmed by the store line in the sequencing abundance curve, the evenness of microbial species, and the platform period of the sob and Shannon curves to meet the requirements of sequencing and analysis. The Simpson index of Yorkshire pig (0.96) was higher than that of the Tibetan pigs (0.92); however, this difference was not significant. The Shannon indices of the two groups were 5.68 and 6.33, respectively, and the difference was not significant. The Chao1 and Sob indices of the Tibetan and Yorkshire pig groups were 1,349.83, 1,396.12, 1,211.17, and 1,261.17, respectively. However, there were no significant differences in the two indices between the groups (*P* > 0.05). The Chao1 and Sob indices showed no significant difference in fungal microbial evenness among the different groups (Figure 4).

### Analysis of Representative Microbial Species of Tibetan and Yorkshire Pigs

**FIGURE 5:**
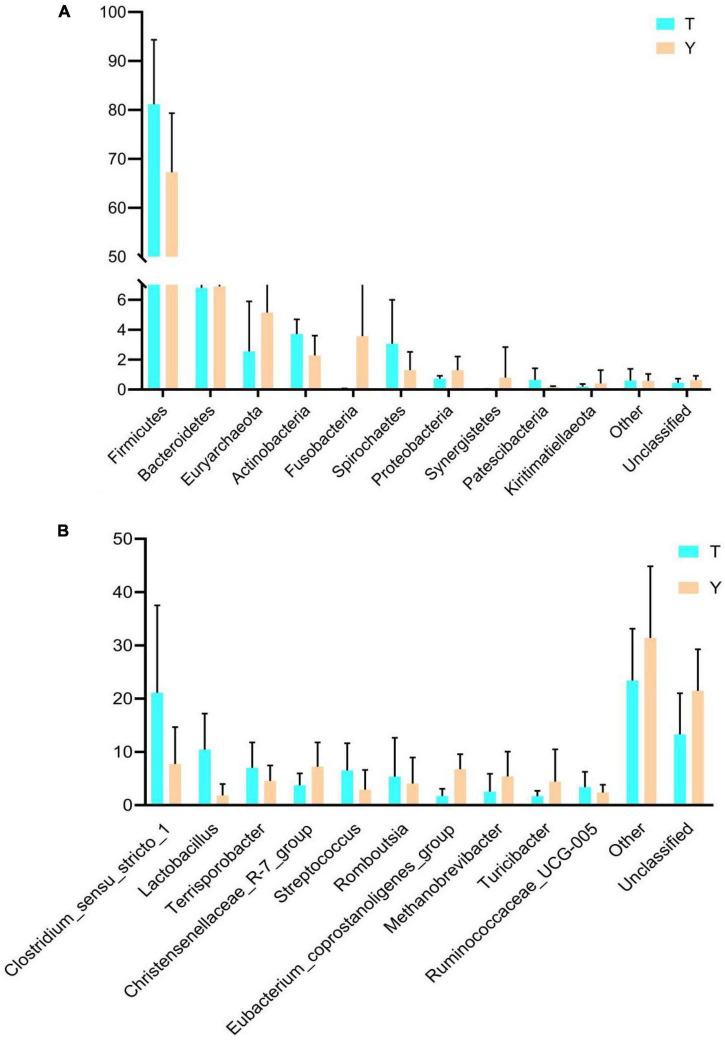
Comparison of community differences in intestinal microbial composition between Tibetan pigs and Yorkshire pigs at the phylum **(A)** and genus **(B)** levels. All data represent average values.

The previous analysis showed that the Tibetan and Yorkshire pigs showed varied colon microbiota at the gate and genus levels; therefore, the microbial community composition of the two levels was analyzed, and the results are shown in Figure 5. The ten most dominant phyla were Firmicutes, Bacteroidetes, Euryarchaeota, Actinobacteria, Fusobacteria, Spirochetes, Proteobacteria, Synergistetes, Patescibacteria, and Kiritimatiellaeota (Figure 5A). Actinomycetes and Spirochetes were the dominant communities in the colons of Tibetan and Yorkshire pigs, accounting for 81.15 and 76.26%, respectively. The relative abundances of Actinomycetes and Spirochetes in Tibetan pigs were significantly higher than those in Yorkshire pigs (*P* < 0.05). There were no significant differences in the relative abundance of other bacteria between Tibetan and Yorkshire pigs (*P* > 0.05). Figure 5B shows great differences in the composition of microflora at the genus level between Tibetan and Yorkshire pigs, and the relative abundance of most microflora in the Tibetan pig colon was higher than that in the Yorkshire pig group. The relative abundance of *Clostridium sensu stricto 1* (21.08%), *Lactobacillus* (10.44%), *Sporobacillus* (6.98%), *Streptococcus* (6.50%), and *Ruminococcaceae*_UCG-005 (3.36%) in the colon microbiota of Tibetan pigs was significantly higher than that in Yorkshire pigs (*Clostridium sensu stricto* 1 7.73%, *Lactobacillus* 1.83%, *Bacillus* 4.53%, *Streptococcus* 2.90%, and *Ruminococcaceae* UCG-005 2.36%) (*P* < 0.05).

**FIGURE 6:**
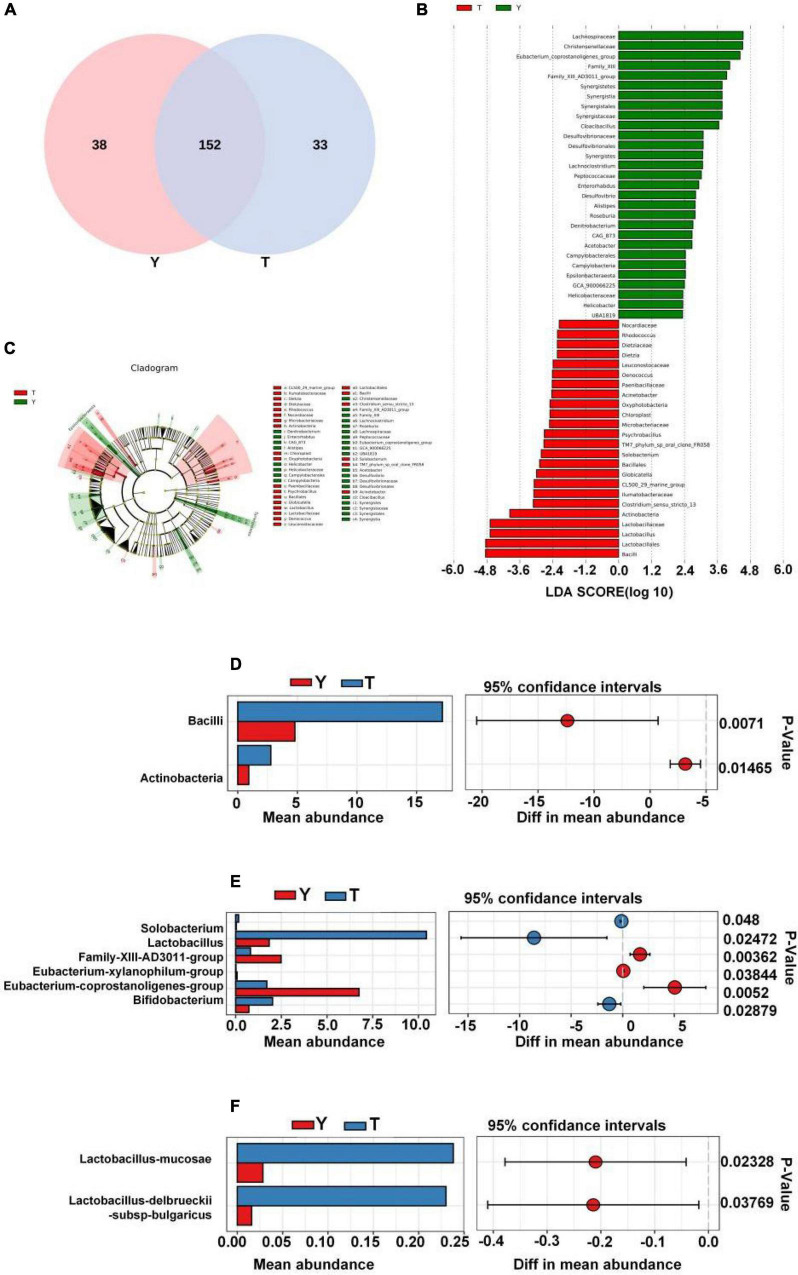
Differences in intestinal microbial composition between Tibetan (T) and Yorkshire (Y) pigs. **(A)** Venn diagram analysis of colonic intestinal microflora in T and Y groups at genus level. **(B)** Variation in abundances of different species between T and Y groups (Linear Discriminant Analysis, LDA > 2). **(C)** Phylogenetic distribution map of microbial communities related to T and Y groups. In the evolutionary tree, the circles from inside to outside represent different levels, and the yellow circles represent taxa with obvious differences. There were significant differences in the class **(D)**, genus **(E)**, and species **(F)** levels of colonic microflora between groups T and Y.

In this study, the Venn diagram of intestinal microorganisms in Tibetan and Yorkshire pigs was intersected at the genus level (Figure 6A). There were 152 genera in these two groups, and 33 species of endemic fungi were found in the colonic secretions of Tibetan pigs. To determine the specific bacterial species in the intestinal microorganisms of Tibetan and Yorkshire pigs, we further analyzed the communities using Linear discriminant analysis Effect Size (LEfSe) with Linear Discriminant Analysis (LDA) > 2, and further determined the differences in species composition between Tibetan and Yorkshire pigs (Figures 6B,C). In the Yorkshire pig group, 29 colons were higher, and 24 were lower in the Tibetan pig group. The 14 OTUs representing bacilli were more abundant in Tibetan pigs. Tibetan pigs contained 10 kinds of OTUs representing *Lactobacillus* (Lactobacillales) and three kinds of OTUs representing Bacillales, both of which belong to the Bacilli class. In addition, Tibetan pigs were enriched in six and two OTUs representing actinomycetes (Actinobacteria) and actinomycetes (Acidimicrobiia), respectively. As shown in Figure 6D, the relative abundance of colonic microbiota in Tibetan pigs in the Bacilli and Actinobacteria classes was significantly higher than that in the Yorkshire pigs (*P* < 0.01 or *P* < 0.05). As shown in Figure 6E, there were extremely significant differences in the compositions of *Solobacterium*, *Lactobacillus*, Family-XIII-AD3011-group, *Eubacterium xylanophilum*-group, *Eubacterium coprostanoligenes*-group, and Bifidobacterium between Tibetan and Yorkshire pigs. The relative abundances of *Solobacterium*, *Lactobacillus*, and *Bifidobacterium* in the colonic microflora of the Tibetan pig group were significantly higher than those of the Yorkshire pig group. As shown in Figure 6F, the relative abundance of *Lactobacillus mucosae* and *Lactobacillus delbrueckii* subsp. *bulgaricus* in the Tibetan pig colonic microbiome was considerably higher than that in the Yorkshire group at the species level, and both *Lactobacillus mucosae* and *Lactobacillus delbrueckii* subsp. *bulgaricus* belonged to the *Lactobacillus* genus.

### Prediction of Ecological Function of Microbiota in Tibetan and Yorkshire Pigs

**FIGURE 7:**
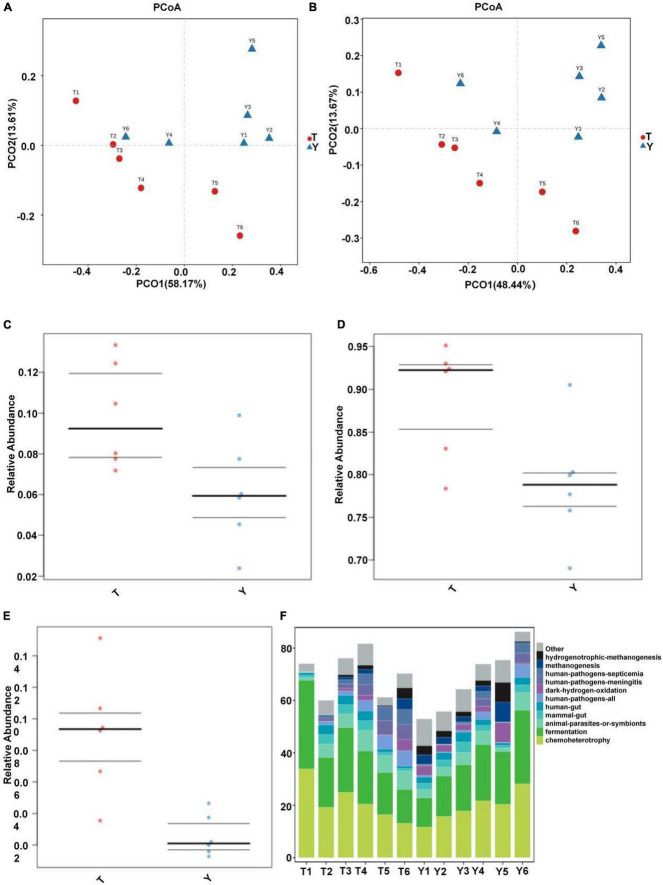
Prediction of ecological functions performed by intestinal microbiota of Tibetan (T) and Yorkshire (Y) pigs. Principal component analysis of group T and group Y at family **(A)** and genus **(B)** levels. Each point represents a sample. Distance between the two points indicates difference in fecal microbiota. Transmembrane transport **(C)**, potential pathogenicity **(D)**, and aerobic activity **(E)** are some of the predicted ecological functions of the intestinal microbiota in both groups **(F)**.

Through principal component analysis, significant differences were observed in fungal structure among the different groups, which was consistent with the previous analysis, especially at the family and genus levels of the Tibetan pig and Yorkshire pig groups (Figures 7A,B). In this study, the abscissa of the stacked chart represents different individuals, and the histogram of different colors in the chart shows the relative abundance of different ecological functions. The microbial communities in groups T and Y were mainly related to metabolism, genetic information processing, cell processes, environmental information processing, organic systems, and human diseases. Its main functions are concentrated in the metabolism of amino acids, cofactors, vitamins, terpenes, holystones, amino acids, and lipids. The comparative abundance of colonic microbial communities in the Tibetan pig group was higher than that in the Yorkshire pig group (Table 2). According to Figures 7C–E, the colonic microbial community of the Tibetan pig group was significantly more active than that of the Yorkshire pigs in the functions of transmembrane transport, potential pathogenicity, and aerobic function (*P* < 0.05) functions. Functional predictions of the top 11 genes are shown in Figure 7F.

**TABLE 2:** Functional prediction of colonic microbiota in Tibetan and Yorkshire pigs.

Level_1	Level_2	*T*	*Y*
Metabolism	Carbohydrate metabolism	305267.04	256121.47
Metabolism	Amino acid metabolism	273214.46	239735.41
Metabolism	Metabolism of cofactors and vitamins	254228.24	227617.69
Metabolism	Metabolism of terpenoids and polyketides	215782.42	182950.76
Metabolism	Metabolism of other amino acids	162799.46	133328.51
Metabolism	Lipid metabolism	156651.76	110977.63
Metabolism	Energy metabolism	116304.73	102474.17
Metabolism	Xenobiotics biodegradation and metabolism	98826.78	74501.90
Metabolism	Glycan biosynthesis and metabolism	58828.02	54900.18
Metabolism	Nucleotide metabolism	48393.17	41063.84
Metabolism	Biosynthesis of other secondary metabolites	43840.67	38691.30
Genetic information processing	Replication and repair	144037.25	120813.52
Genetic information processing	Translation	77198.95	70203.12
Genetic information processing	Folding, sorting and degradation	71196.89	63858.14
Genetic information processing	Transcription	22587.97	24442.64
Cellular processes	Cell motility	47364.51	54467.32
Cellular processes	Cell growth and death	34557.93	28240.98
Cellular processes	Transport and catabolism	4270.04	3029.48
Cellular processes	Cellular community - prokaryotes	3505.88	3274.08
Environmental information processing	Membrane transport	50752.30	36982.53
Environmental information processing	Signal transduction	8003.29	7446.62
Environmental information processing	Signaling molecules and interaction	0.62	0.64
Organismal systems	Environmental adaptation	4677.55	4643.96
Organismal systems	Endocrine system	2051.06	1907.85
Organismal systems	Immune system	1372.58	1426.66
Organismal systems	Digestive system	257.11	204.33
Organismal systems	Excretory system	0.01	0.01
Human diseases	Infectious diseases	7123.71	4664.47
Human diseases	Neurodegenerative diseases	380.00	250.24
Human diseases	Cardiovascular diseases	0.43	0.81
Human diseases	Immune diseases	0.01	0.00

## Discussion

The pig is commercially important in animal husbandry, and an important biomedical model of human beings. The number of pigs in the world is estimated to be approximately 1 billion. Intestinal microbes can regulate the growth characteristics and health status of the host, such as fat deposition traits (Lei et al., 2021), chronic diseases (cancer and metabolic disorders) (Coleman et al., 2018; Just et al., 2018; Zitvogel et al., 2018), and disease resistance (resistance to intestinal infection) (Kumar et al., 2018). The Tibetan pig is a unique and valuable pig breed from the Tibetan Plateau that shows strong fat deposition ability, strong disease resistance, adaptability to high altitude hypoxia, and resistance to the cold and rough feeding.

Intestinal morphology is very important for nutrient digestion and absorption, and intestinal villus length, goblet cell characteristics, mucosal thickness, and muscle thickness are integral for this function. In general, dietary fiber intake leads to an increase in the size and length of the digestive organs, such as the cecum and colon of pigs, chicken, and rats. These effects are usually associated with changes in the morphology of intestinal epithelial cells, thus affecting the hydrolysis and absorption function of epithelial cells (Hedemann et al., 2006). The intestinal mucosa, muscle thickness, and intestinal microorganisms are closely related to the disease resistance of animals, and they interact to maintain the health of animals (Candela et al., 2008). The results showed that the mucosal and muscle layer thicknesses of Tibetan pigs were higher than those of Yorkshire pigs. This may be because Tibetan pigs eat more crude fiber food and show outstanding disease resistance, which are consistent with previous studies.

The pig intestine is a microenvironment composed of numerous microflora, which is generally regarded as a large metabolic spectrum that maintains its basic life and has a considerable impact on the growth and health of the host by participating in energy, metabolism, the intestinal barrier, and immune function (Langille et al., 2013; Lo et al., 2021). Among them, the relationship between microorganisms and microorganisms, between microorganisms and the intestinal environment, and between microbial communities and hosts constitutes an extremely complex ecosystem in which the main composition of the microbial community is a thick-walled phylum and Bacteroides (Tan et al., 2017; Lu et al., 2018). The results showed a relative abundance of actinomycetes. *Clostridium* and *Spirochetes* were higher in the colonic microorganisms of Tibetan and Yorkshire pigs, which is consistent with the results of previous studies.

The composition of the intestinal microbial community greatly influences health. The intestinal microbiota is very important for nutrition, energy, inflammatory immunity, and physiological status of pigs. Simultaneously, the breed, age, body weight, diet, heredity, environment, and other factors cause changes in the intestinal microflora (Yang et al., 2017; Crespo-Piazuelo et al., 2019; Wang et al., 2019). The relative abundance of colonic microbial communities in the Tibetan pig group was higher than that in the Yorkshire pig group. This may be explained by the fact that Yorkshire pigs were raised in houses, whereas Tibetan pigs are fed in semi-houses. Additionally, Tibetan pigs also ate grass, tree roots, grass roots, and insects. Studies have demonstrated that a high-fiber diet can promote the diversity of the intestinal flora; therefore, intestinal microorganisms were more diverse in Tibetan pigs than that in Yorkshire pigs. This is in agreement with the study by Ngoc et al. (2021), which showed that dietary fiber has significant effects on the intestinal environment and microflora of pigs. Pig breeds and different diets can cause significant changes in the ideal colonic microflora. The Tibetan pig is a fat pig breed, while the Yorkshire pig is a typical lean pig breed. Intestinal microorganisms not only provide energy for life-sustaining activities, but are also involved in regulating lipid storage (Backhed et al., 2004). Additionally, the abundance of intestinal microflora is significantly correlated with obesity parameters (Bergamaschi et al., 2020; Hao et al., 2021). Colonic microbes and complex traits such as obesity have been shown to be closely related (Backhed et al., 2004; Camarinha-Silva et al., 2017) in humans, mice, and other animals. The data show that the aseptic mice colonized by the microbiota of obese mice showed more body fat (Turnbaugh et al., 2006) than lean mice, which provides credibility for the role of intestinal microflora in obesity. Therefore, obesity and fat deposition traits of pigs may also be one of the reasons for the difference in abundances between colonic microbial communities in Tibetan and Yorkshire pigs.

The colon is the primary site for microbial fermentation, and the core intestinal flora directly affects intestinal function (Luo et al., 2021). Tibetan pigs live in high-altitude and cold-plateau environments year-round, exhibiting plateau adaptability, resistance to the cold and rough feeding, and stress resistance. *Chlamydia* was found to be the dominant microflora in the colonic microflora of Tibetan pigs (semi-house feeding) and large York pigs (house feeding), and the relative abundance of Actinomycetes and *Spirulina* in Tibetan pigs was significantly higher than that of Yorkshire pigs. The ratio of Actinomycetes to Bacteroides in Tibetan pigs (11.93) was slightly higher than that in Yorkshire pigs (11.84). The changes in the abundance of Bacteroides and Bacteroides are related to changes in carcass fat deposition (Pedersen et al., 2013). In the core intestinal microbiome of obese and lean twins, *Chlamydia*/*Pseudomonas* ratio was associated with greater energy absorption and accumulation (Turnbaugh et al., 2009). Some studies further showed that the abundance of *Streptomyces* and *Streptomyces* was higher in the intestinal microbiota of obese pigs, whereas that of *Bacteroides* was lower (Guo et al., 2008; Koliada et al., 2017; Panasevich et al., 2018). At the genus level, the relative abundance of *Bifidobacterium*, *Lactobacillus*, Family-XIII-AD3011-group, *Ruminococcaceae* UCG-005, and *Solobacterium* was greater than that in Yorkshire pigs. *Bifidobacterium*, an actinomycete, is a gram-positive bacterium that acts as an indicator of intestinal health, and can maintain the balance of intestinal microecology. *Bifidobacteria* can inhibit the reproduction of harmful microorganisms by forming intestinal biological barriers, producing organic acids and germicidal proteins, and secreting extracellular glycosidases. *Bifidobacteria* can also synthesize various digestive enzymes. Vitamin B and amino acids promote the digestion and absorption of nutrients (Binda et al., 2018; Wong et al., 2020). *Lactobacillus* plays an important role in metabolizing plant foods (Filannino et al., 2018) and participates in producing some antimicrobials with anticancer and anti-inflammatory effects (Fernández et al., 2016). Wang et al. (2017) reported that *Lactobacillus* was associated with growth and fat deposition traits in broilers. Some studies have screened individual microorganisms that play a critical role in the substantial effects of the cecum, colon, and jejunum on growth and fat-related traits in pigs. Among the 10 microorganisms screened, nine were located in the cecum and colon, indicating that the cecum and colon play a more important role than the jejunum, and *Ruminococcaceae* UCG-005 in the colon showed a highly positive correlation with body weight and average daily gain (Tang et al., 2020). They are widely present in different intestinal communities and can degrade plant polysaccharides (Biddle et al., 2013). They can also produce butyric acid and acetic acid (Vital et al., 2014) *via* the butyryl-coenzyme A (CoA): acetic acid CoA transferase pathway. Butyric acid is the main energy source for colonic mucosal epithelial cells, which can maintain the structural integrity of the intestinal mucosa and promote the growth of the large intestine. Butyrate also has a powerful effect on a variety of colonic mucosal functions, such as inhibiting inflammation and carcinogenicity and strengthening various components of the colon defense barrier (Peng et al., 2009). The reduction in short-chain fatty acids produced by intestinal microorganisms can lead to inflammation (Maslowski and Mackay, 2011). In addition, higher concentrations of short-chain fatty acids (Payne et al., 2011) were found in obese individuals. *Ruminococcaceae* UCG-005 also benefits hosts by preventing diabetes and increasing colonic levels of short-chain fatty acids (Andrade et al., 2020). In a study on goats, Wang et al. (2018) found that the abundance of *Ruminococcaceae* UCG-005 in the intestinal tract of kids with diarrhea was significantly lower than that in healthy goat kids. *Ruminococcaceae* and Family_XIII are members of Clostridium. The results showed that the relative abundance of *Bifidobacterium*, *Lactobacillus*, *Ruminococcaceae* UCG-005, and Family-XIII-AD3011-group in the colon of Tibetan pigs was higher than that in Yorkshire pigs, indicating that the fat deposition, intestinal health, and disease resistance of Tibetan pigs were higher than those of Yorkshire pigs. The main functions of colonic microflora in Tibetan and Yorkshire pigs are concentrated in the metabolism of amino acids, cofactors and vitamins, terpenes and holystones, and amino acids and lipids. For all functions, the comparative abundance of the colonic microbial community in the Tibetan pig group was higher than that in the Yorkshire pig group. This is consistent with the fact that fat deposition and disease resistance in Tibetan pigs are higher than in Yorkshire pigs.

## Conclusion

This study investigated the effects of colonic microbial communities, fat deposition traits, and disease resistance in Tibetan pigs. The relative abundance of colonic microflora in Tibetan pigs was higher than that of Yorkshire pigs. Particularly, the relative abundances of *Bifidobacterium*, *Ruminococcaceae*, and Family-XIII-AD3011-group in Tibetan pigs were significantly higher than that in Yorkshire pigs, which is the major microbial group responsible for the disease resistance of Tibetan pigs. The relative abundance of *Lactobacillus* and *Solobacterium* in Tibetan pigs was significantly higher than that in Yorkshire pigs, which mainly affected the fat deposition traits of Tibetan pigs. This study will provide a new reference for future development and utilization of Tibetan pigs.

## Data Availability Statement

The data presented in this study are deposited in the NCBI repository, accession number PRJNA848282.

## Ethics Statement

The animal study was reviewed and approved by the rearing, slaughtering and experimental conditions for experimental were strictly followed the guidelines approved by the Animal Welfare Committee of the Tibet Agriculture and Animal Husbandry University (Approval Number: TAAHU256).

## Author Contributions

PS and YC conceived and designed the experiments. MW and MD analyzed the data. MW, PS, YC, and FY provided manuscript editing. All authors statistically analyzed, discussed, critically revised the contents, and approved the final manuscript.

## Conflict of Interest

The authors declare that the research was conducted in the absence of any commercial or financial relationships that could be construed as a potential conflict of interest.

## Publisher’s Note

All claims expressed in this article are solely those of the authors and do not necessarily represent those of their affiliated organizations, or those of the publisher, the editors and the reviewers. Any product that may be evaluated in this article, or claim that may be made by its manufacturer, is not guaranteed or endorsed by the publisher.

## Footnotes

1
https://github.com/OpenGene/fastp

2
http://drive5.com/uchime/uchime_download.html

3
http://www.drive5.com/usearch/manual/uchime_algo.html

4
https://www.arb-silva.de/

## Abbreviations

OUT: operational taxa.
